# Reducing the Activity and Secretion of Microbial Antioxidants Enhances the Immunogenicity of BCG

**DOI:** 10.1371/journal.pone.0005531

**Published:** 2009-05-13

**Authors:** Shanmugalakshmi Sadagopal, Miriam Braunstein, Cynthia C. Hager, Jie Wei, Alexandria K. Daniel, Markian R. Bochan, Ian Crozier, Nathaniel E. Smith, Hiriam O. Gates, Louise Barnett, Luc Van Kaer, James O. Price, Timothy S. Blackwell, Spyros A. Kalams, Douglas S. Kernodle

**Affiliations:** 1 Department of Medicine, Vanderbilt University Medical Center, Nashville, Tennessee, United States of America; 2 Department of Microbiology and Immunology, University of North Carolina, Chapel Hill, North Carolina, United States of America; 3 Department of Microbiology and Immunology, Vanderbilt University Medical Center, Nashville, Tennessee, United States of America; 4 Department of Veterans Affairs Medical Center, Nashville, Tennessee, United States of America; University of Hyderabad, India

## Abstract

**Background:**

In early clinical studies, the live tuberculosis vaccine *Mycobacterium bovis* BCG exhibited 80% protective efficacy against pulmonary tuberculosis (TB). Although BCG still exhibits reliable protection against TB meningitis and miliary TB in early childhood it has become less reliable in protecting against pulmonary TB. During decades of in vitro cultivation BCG not only lost some genes due to deletions of regions of the chromosome but also underwent gene duplication and other mutations resulting in increased antioxidant production.

**Methodology/Principal Findings:**

To determine whether microbial antioxidants influence vaccine immunogenicity, we eliminated duplicated alleles encoding the oxidative stress sigma factor SigH in BCG Tice and reduced the activity and secretion of iron co-factored superoxide dismutase. We then used assays of gene expression and flow cytometry with intracellular cytokine staining to compare BCG-specific immune responses in mice after vaccination with BCG Tice or the modified BCG vaccine. Compared to BCG, the modified vaccine induced greater IL-12p40, RANTES, and IL-21 mRNA in the spleens of mice at three days post-immunization, more cytokine-producing CD8+ lymphocytes at the peak of the primary immune response, and more IL-2-producing CD4+ lymphocytes during the memory phase. The modified vaccine also induced stronger secondary CD4+ lymphocyte responses and greater clearance of challenge bacilli.

**Conclusions/Significance:**

We conclude that antioxidants produced by BCG suppress host immune responses. These findings challenge the hypothesis that the failure of extensively cultivated BCG vaccines to prevent pulmonary tuberculosis is due to over-attenuation and suggest instead a new model in which BCG evolved to produce more immunity-suppressing antioxidants. By targeting these antioxidants it may be possible to restore BCG's ability to protect against pulmonary TB.

## Introduction

Oxidants fulfill signaling roles during the activation of innate immune responses and promote the development of adaptive immunity [1]–[3]. Growing evidence implicates microbial antioxidants in the suppression of host immune responses to *Mycobacterium tuberculosis*. The iron co-factored superoxide dismutase (SodA) and its secretion channel, SecA2, suppress macrophage activation in vitro and inflammatory and immune responses in vivo [4]–[6]. An extracytoplasmic-function sigma factor, SigH, controls the expression of several antioxidants including thioredoxin and is associated with lung immunopathology [7]–[9]. Other antioxidants including catalase-peroxidase, alkylhydroperoxidase C, and the NADPH quinone reductase Rv3303c also contribute to mycobacterial virulence [10]–[12].

The current vaccine against tuberculosis (TB), the bacillus of Calmette and Guérin (BCG), also secretes large amounts of antioxidants. BCG is widely administered however it has failed to control the enormous global burden of TB that causes about 9 million new active cases and 1.7 million deaths annually [13]–[16]. BCG is a live vaccine derived from *M. bovis* via the loss of RD1, the “region of deletion 1” that encodes the ESX-1 secretion system involved in virulence [17]. During the first half of the 20^th^ century BCG was sent to investigators throughout the world and maintained by serial passage, *i.e*., by transferring a portion of an aging culture into fresh media. Over decades this practice produced multiple BCG daughter strains with additional regions of genomic deletion as well as regions of genomic duplication and other mutations [17]–[19]. These substrains represent divergent evolution from the original BCG and some have been transferred over 1500 times [17]. Some BCG daughter strains exhibit genomic duplication of *sigH, trxC* (thioredoxin), *trxB2* (thioredoxin reductase), *whiB1, whiB7,* and *lpdA* (*Rv3303c*) as well as increased expression of genes encoding other antioxidants including SodA, thiol peroxidase, alkylhydroperoxidases C and D, and other members of the whiB family of thioredoxin-like protein disulfide reductases [17], [19]–[21].

Although attenuated from the loss of RD1, some BCG substrains exhibit potent immune suppressive properties. BCG inhibits phagolysosomal fusion in macrophages [22], reduces monocyte apoptosis [23], down-regulates MHC class II molecules [24], and suppresses the maturation of dendritic cells [25]. The immune suppressive capacity of BCG is perhaps most apparent in vivo. Some BCG daughter strains persist indefinitely in small animals [4], [26]–[28], induce lung pathology [4], [29], and cause death when administered as a large dose [30]. In man, the rising prevalence of HIV infection and virulence attributes of BCG have raised concerns about disseminated BCG infection after routine neonatal vaccination and it is estimated that more than 2000 serious complications of BCG vaccination occur annually [31].

To test the hypothesis that the duplication of SigH and production of SodA contribute to the immune suppressive capacity of BCG, we modified an extensively cultivated BCG daughter strain to eliminate both copies of *sigH* and to reduce the activity and secretion of SodA. The modified vaccine induced stronger immune responses than the parent BCG vaccine during primary vaccination as well as enhanced recall immune responses upon subsequent challenge. These observations have important implications for efforts to develop a more effective live vaccine against TB. They further suggest the novel hypothesis that the extensively cultivated BCG daughter strains are immune suppressive as a consequence of increased antioxidant production. This contrasts with the theory that the extensively cultivated BCG daughter strains are over-attenuated although both theories attempt to explain the apparent decline in efficacy of BCG against pulmonary TB during decades of cultivation in vitro [32], [33]. Finally, the findings highlight the importance of host-generated oxidants in the development of adaptive immunity.

## Results

### Construction and in vitro characterization of 3dBCG, a modified BCG vaccine

**Figure 1:**
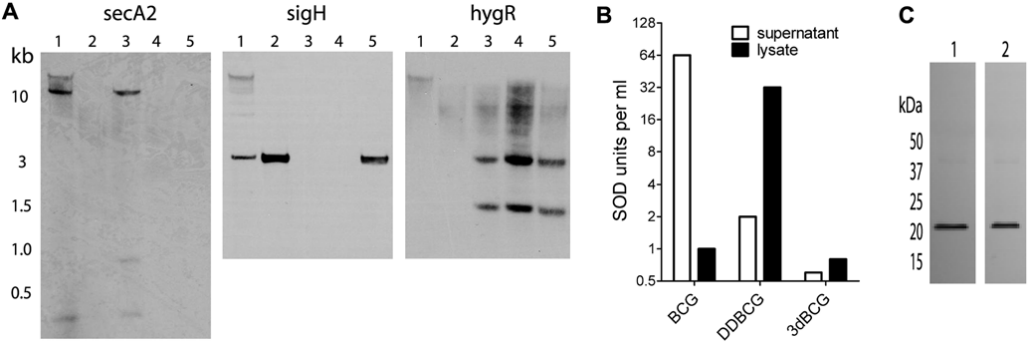
Construction of modified BCG vaccines. (A) Southern hybridization demonstrating inactivation of *secA2* and *sigH*. *DraIII*-digested DNA from each strain was probed for *secA2*, *sigH* and the *hygR* cassette used to inactivate *sigH*. Lane 1, BCG Tice; lane 2, BCGΔ*secA2*; lane 3, BCGΔ*sigH*; lane 4, BCGΔ*secA2*Δ*sigH* (DDBCG); lane 5, a colony of BCGΔ*secA2* with only one copy of *sigH* inactivated. *secA2* contains two internal *DraIII* sites and *DraIII*-digested DNA was predicted to yield fragments of 307 bp, 785 bp, and 10,544 bp. *sigH* was predicted to be on a 2895 bp fragment. The *hygR* cassette contains an internal *DraIII* site and was predicted to yield 2938 bp and 1612 bp fragments after inactivation of *sig*H. (B) Differences in the localization and activity of SodA in BCG, DDBCG, and 3dBCG. A representative experiment shows the SOD units per ml of concentrated supernatant or lysate. (C) Immunoblot showing comparable amounts of SodA in lysates of DDBCG (lane 1) and 3dBCG (lane 2), despite the marked difference in SOD activity as shown in (b).

We used allelic inactivation to eliminate *secA2* and *sigH* in BCG Tice, and dominant-negative (dn) interference techniques to reduce the activity of SodA. To construct BCGΔ*secA2*Δ*sigH* (“double-deletion BCG”, “DDBCG”) *secA2* and *sigH* were inactivated in sequence (**Fig. 1A**). During attempts to inactivate *sigH* with a hygromycin-resistance (*hygR*) cassette we found that only about 15% of colonies lacked *sigH* whereas most colonies contained an intact *sigH* along with DNA restriction fragments suggesting successful interruption of *sigH* by the *hygR* allele. This finding is consistent with reports that most BCG daughter strains have two copies of *sigH* due to tandem duplication of the region of chromosomal DNA containing *sigH* [17], [19].

After verifying that *sigH* was absent from the colony selected for further modification, we inserted an allele encoding a dominant-negative mutant of SodA into the chromosome of DDBCG to yield 3dBCG (BCGΔ*secA2*Δ*sigH*dnSodA). SodA contains histidines that chelate the active-site iron [34] and we eliminated H28 and H76 to produce an inactive SodA mutant. Enzyme activity assays (**Fig. 1B**) and immunoblotting (**Fig. 1C**) demonstrated a dominant-negative effect with reduced enzyme activity in 3dBCG compared to DDBCG despite comparable amounts of SodA protein, presumably because ΔH28ΔH76 monomers were incorporated into the tetrameric, functional form of SodA and inhibited enzyme activity. Compared to BCG, the inactivation of secA2 in DDBCG nearly eliminated SodA secretion however enzyme activity was present in the lysate. Compared to DDBCG, 3dBCG exhibited a 95% reduction in enzyme activity. In summary, we introduced three modifications into BCG to reduce antioxidant production, yielding 3dBCG.

### 3dBCG induces stronger recall T cell responses

Most vaccines are administered to generate memory lymphocytes that can proliferate rapidly, thereby accelerating the kinetics and magnitude of immune responses to a subsequent infection. To determine whether vaccination with 3dBCG or BCG induce recall immune responses, we immunized C57Bl/6 mice subcutaneously with BCG, 3dBCG, or sterile media (control vaccination group). BCG typically can be recovered months after inoculation of C57Bl/6 mice depending upon the substrain, dose, and route of administration [4], [26], [27], however BCG-induced T cell responses decline rapidly almost to pre-vaccination levels within a few months of treating vaccinated mice with antibiotics [35]. Thus, to assess recall immunity without interference from persisting vaccine bacilli, we treated the vaccinated groups of mice with isoniazid and rifampin from day 30 to day 60 post-vaccination and this reduced the spleen titer of vaccine bacilli to below the lower limits of detection (50 CFU). After resting the mice for an additional 2 to 9 weeks, they were challenged intravenously with 2×10^7^ CFU of BCG Tice.

**Figure 2:**
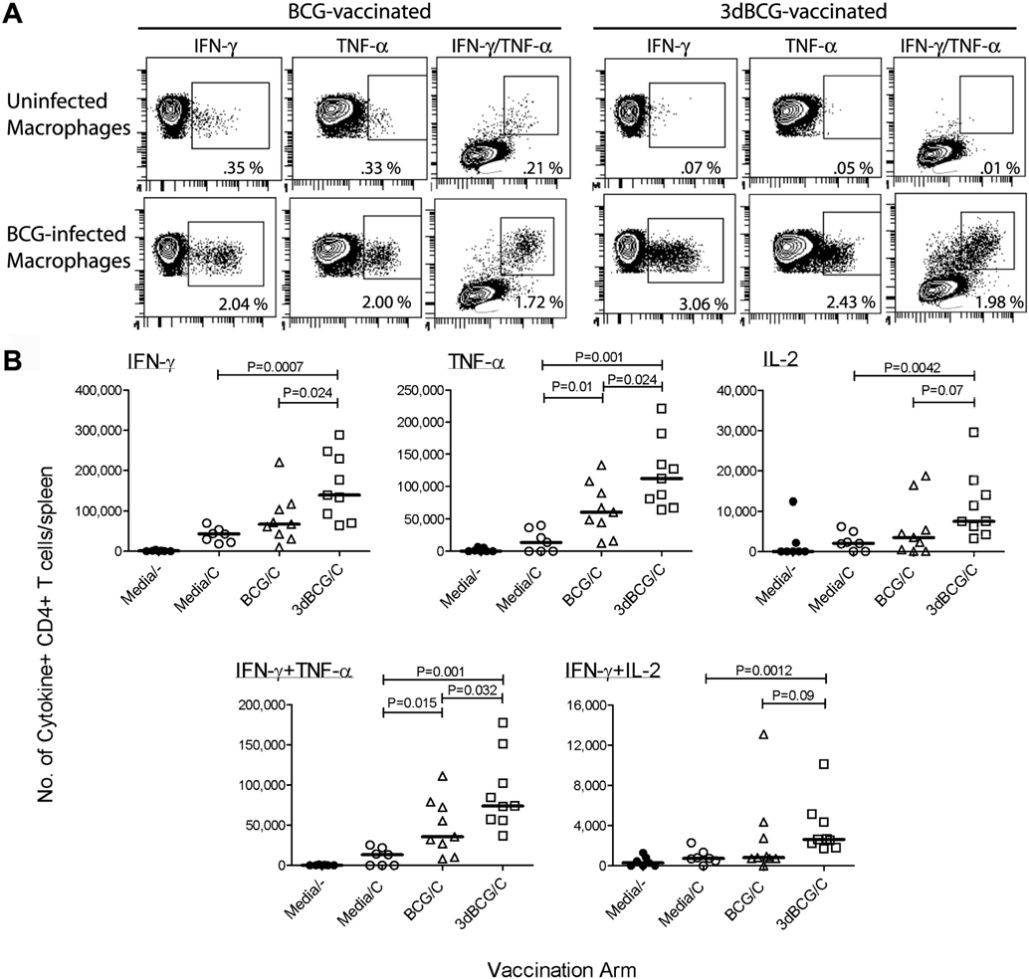
Cytokine-producing CD4+ T-cells in the spleens of vaccinated mice after intravenous challenge. (A) Representative plots show IFN-γ+, TNF-α+ and IFN-γ+TNF-α double-positive cytokine production by splenocytes of mice vaccinated subcutaneously with BCG or 3dBCG at 28 days after intravenous challenge with 2×10^7^ CFU of BCG. Splenocytes were re-stimulated on IFN-γ-treated uninfected bone-marrow derived macrophages and BCG-infected macrophages. Percent values represent frequency of cytokine+ CD4+ T cells for each stimulation condition. (B) Number of IFN-γ+, TNF-α+, IL-2+ and dual cytokine+ CD4+ T cells in spleens 26 to 30 days after intravenous challenge with BCG. Each data point represents the number of BCG-specific cytokine+ T cells in one mouse spleen, calculated from percent values of cytokine+ CD4+ T cells, as in (a), and total number of splenocytes isolated from each mouse. The bars represent median values from 7 to 9 mice in each vaccinated group. Media/- mice are age-matched controls that were vaccinated with media but not challenged, C = challenged. The variances of the median values of the three challenged groups were compared by the Kruskal-Wallis non-parametric test: IFN-γ, P = 0.0031; TNF-α, P = 0.0006; IL-2, P = 0.0165; IFN-γ+TNF-α, P = 0.0007; and IFN-γ+IL-2, P = .0131. The Mann-Whitney P values for two group comparisons are displayed in the figure.

Recall immunity was evaluated on days 26 to 30 post-challenge by incubating splenocytes on uninfected and BCG-infected bone marrow-derived macrophages (BMDMs). Antigen-specific cytokine production, quantified by intracellular cytokine staining and flow cytometry (**Fig. 2A**), was reported as the number of cytokine+ T cells per spleen. The magnitude of the CD4+ IFN-γ T cell response following challenge in BCG-vaccinated mice (median 67,560/spleen) was not significantly greater than in media-vaccinated control mice (median 43,300/spleen) undergoing a primary response to the BCG challenge (**Fig. 2B**). In contrast, more IFN-γ+ CD4+ T cells were present in 3dBCG-vaccinated mice (median 138,500/spleen). The number of CD4+ lymphocytes producing TNF-α or both IFN-γ and TNF-α was also greater in 3dBCG-vaccinated mice than in BCG-vaccinated or control mice.

The production of IL-2 is associated with multi-functional, high-quality memory T cells [36] and thus as a secondary endpoint we also evaluated the IL-2 recall response. The number of IL-2+ and IFN-γ+IL-2+ (double-positive) CD4+ T cells in BCG-vaccinated mice was not significantly greater than the response of control mice (**Fig. 2B**). In contrast, more IL-2+ and IFN-γ+IL-2+ CD4+ T cells were present in 3dBCG-vaccinated mice than in control mice. For the comparisons involving 3dBCG- and BCG-vaccinated mice, there was a trend towards statistical significance (P>0.05, <0.1). The numbers of IFN-γ- or TNF-α-producing CD8+ T cells in the spleen were low at day 26 to 30 post-challenge (data not shown) compared to the number of CD4+ T cells producing these cytokines. However, five of nine 3dBCG-vaccinated mice exhibited greater than 10,000 IL-2+ CD8+ T cells in the spleen compared to none of seven media-vaccinated mice (P = 0.03, Fisher's exact, two-tailed) and one of nine BCG-vaccinated mice.

**Figure 3:**
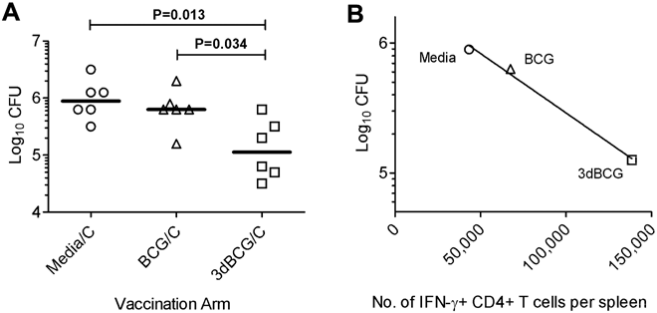
Spleen mycobacterial titers post-challenge and correlation between bacilli counts and T cell responses. (A) BCG colony-forming units (CFU) persisting in the spleens of mice on day 26–30 after intravenous challenge with 2×10^7^ CFU of BCG. Data are from six mice per group from two experiments. Individual and median values are displayed. The variance of the medians of the three groups was significant, P = 0.0186, Kruskal-Wallis test. Mann-Whitney P values for two-group comparisons are shown in the figure. (B) Plot of median Log_10_ CFU in spleens of vaccinated mice versus the median CD4+ IFN-γ responses for the three vaccination arms. Coefficient of correlation, R^2^ was calculated using median values from each vaccination group.

After noticing higher cytokine production in re-stimulated splenocytes of 3dBCG-vaccinated mice in the first experiment, we enumerated challenge bacilli in spleens of mice in subsequent experiments and thus determined post-challenge CFU values for six vaccinated mice per group. Fewer bacilli of the BCG challenge dose were found in the spleens of 3dBCG-vaccinated mice than in BCG-vaccinated mice (median Log_10_ CFU of 5.05 and 5.80, respectively, P = 0.034) or media-vaccinated mice (median Log_10_ CFU 5.95, P = 0.013) (**Fig. 3A**). An inverse correlation between median CFU and median CD4+ IFN-γ T cell responses from each of the three vaccinated groups was observed (R^2^ = 0.99) (**Fig. 3B**), indicating that the stronger 3dBCG-induced recall CD4+ IFN-γ responses were associated with better clearance of the challenge bacilli. Therefore, under circumstances in which the live vaccine strain is not allowed to persist, prior vaccination with 3dBCG enhanced BCG-specific immune responses post-challenge and reduced the number of challenge bacilli, whereas prior vaccination with BCG did not confer much advantage over unvaccinated mice.

### 3dBCG induces stronger CD8+ T cell responses during primary vaccination

**Figure 4:**
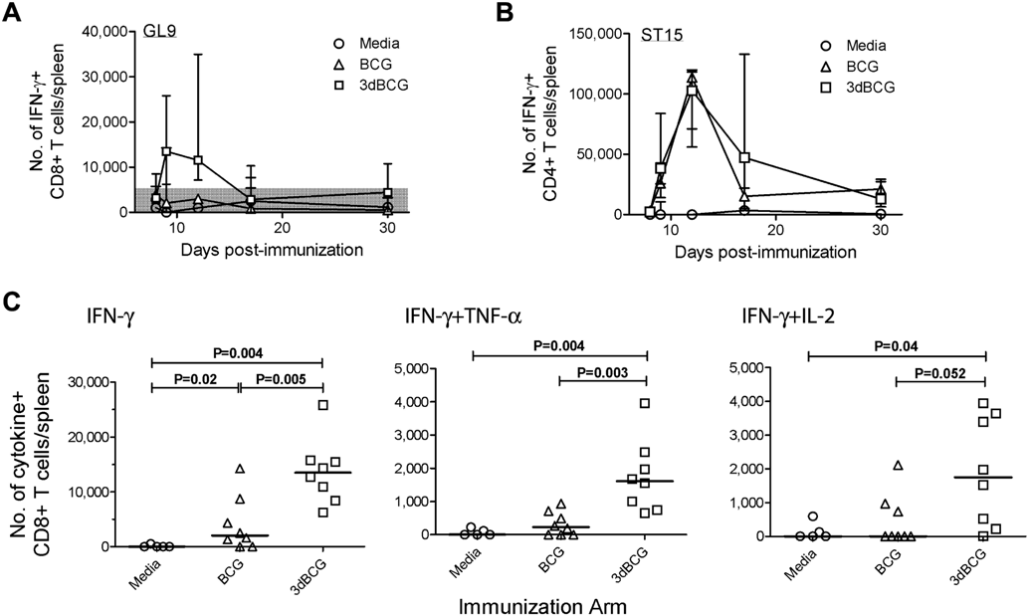
Kinetics of the primary immune response and peak CD8+ T cell responses. (A) Numbers of IFN-γ+ CD8+ T-cells in spleens after re-stimulation with TB10.3/10.4_20-28_ peptide (GL9) at 8, 9, 12, 17 and 30 days after intravenous inoculation of media, BCG or 3dBCG. The symbols and error bars represent median and range of responses of 3 to 9 mice per time point. (B) Numbers of IFN-γ+ CD4+ T cells in spleens after re-stimulation with TB10.4_74-88_ peptide (ST15) on days after vaccination. (C) Numbers of IFN-γ+, and IFN-γ+TNF-α+, and IFN-γ+IL-2+ CD8+ T cells in spleens after re-stimulation with TB10.3/10.4_20-28_ peptide - GL9 at the peak of the primary CD8+ T cell response (day 9 post-inoculation). Each symbol represents the number of cytokine-producing CD8+ T cells from one mouse. The bars represent median values for each group and Mann-Whitney P values for two-group comparisons are displayed in the figure. The variances of the medians of the three groups were significant: IFN-γ, P = 0.0009; IFN-γ+TNF-α, P = 0.0013; and IFN-γ+IL-2, P = 0.0415, Kruskal-Wallis test.

Results from the recall immune response experiments above led us to evaluate primary T cell responses in more detail. We used Balb/c mice to track peptide-specific responses to immunodominant epitopes including TB10.4_74-88_ - ST15, a CD4 epitope [37], and TB10.3/10.4_20-28_ - GL9, a CD8 epitope [38]. Preliminary experiments showed comparable numbers of cytokine-producing CD4+ T cells in mice vaccinated subcutaneously and intravenously, however the intravenous group demonstrated greater CD8+ T cell responses by flow cytometry (data not shown). Thus, to determine the kinetics and peak of the primary response we inoculated Balb/c mice intravenously with 5×10^5^ CFU of BCG or 3dBCG and analyzed the number of IFN-γ-producing CD8+ and CD4+ T cells at 8, 9, 12, 17 and 30 days post-inoculation. The CD8+ T cell response to peptide GL9 peaked at day 9 post-inoculation **(** **Fig. 4A** **)** and the CD4+ T cell response to peptide ST15 peaked at day 12 **(** **Fig. 4B** **)**. The kinetics of the T cell responses to BCG and 3dBCG vaccination were similar however vaccination with 3dBCG induced higher peak IFN-γ+, IFN-γ+TNF-α+ and IFN-γ+IL-2+ CD8+ T cell responses to GL9 than vaccination with BCG **(** **Fig. 4C** **)**.

### 3dBCG induces more IL-2-producing CD4+ T cells

**Figure 5:**
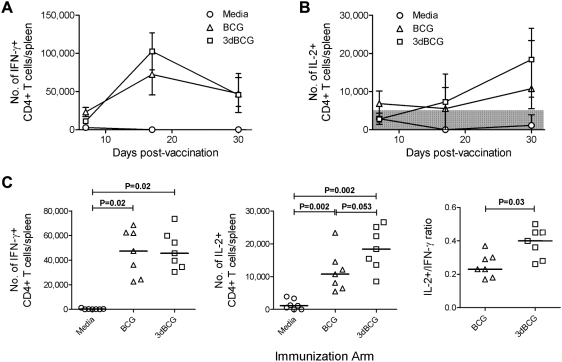
Memory CD4+ T cell responses after subcutaneous vaccination with BCG or 3dBCG. Kinetics of (A) IFN-γ+ and (B) IL-2+ CD4+ T cell response in spleens of subcutaneously vaccinated mice in response to TB10.4_74-88_ peptide - ST15 stimulation. The symbols represent median and range of cytokine responses of 3–7 mice per time point. (C) Numbers of IFN-γ+ and IL-2+ CD4+ T cells in spleens and the IL-2+/IFN-γ+ ratio of CD4+ T cells in each spleen after re-stimulation with TB10.4_74-88_ peptide - ST15 at the memory phase of the primary CD4+ T cell response (day 30 post-vaccination). Each data point represents the number of cytokine+ CD4+ T cells or the ratio from one mouse, with 7 mice per group. The bars represent median values for each group and Mann-Whitney P values for two-group comparisons are displayed in the figure. The variances of the medians of the three groups were significant: IFN-γ, P = 0.0012; and IL-2, P = 0.0005, Kruskal-Wallis test.

The presence of IL-2 during the contraction phase of the primary immune response is critical for the development of memory T cells that can subsequently proliferate to provide a recall T cell response [36], [39], [40]. The kinetics of the immune response after subcutaneous vaccination of Balb/c mice with 5×10^5^ CFU of BCG or 3dBCG demonstrated declines in the number of IFN-γ+ CD4+ T cells and increases in the number of IL-2+ CD4+ T cells in response to peptide ST15 from day 17 to day 30 post-vaccination **(** **Fig. 5A,B** **)**. The number of ST15-specific IFN-γ- or TNF-α-producing CD4+ T cells at the peak (day 17) or memory phase (day 30) CD4+ T cell response were similar in the BCG and 3dBCG groups. In contrast, vaccination with 3dBCG induced more ST15-specific IL-2+ CD4+ T cells during the memory phase (median 18,400/spleen) than vaccination with BCG (median 10,760/spleen) **(** **Fig. 5C** **)**. The higher ratio of IL-2+ to IFN-γ+ CD4+ T cells in 3dBCG-vaccinated mice compared to BCG-vaccinated mice may have contributed to the greater recall immune response in 3dBCG-vaccinated mice during subsequent challenge.

### 3dBCG induces greater early expression of IL-12p40, RANTES, and IL-21 mRNA

**Figure 6:**
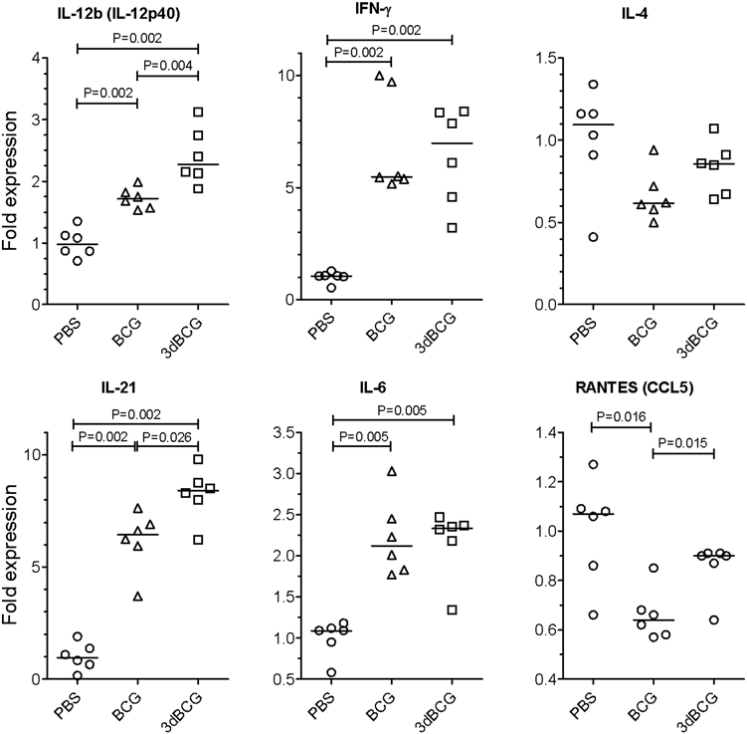
Expression of genes encoding Th polarizing cytokines. Mice were inoculated by the retro-orbital route and spleens harvested 72 hours later. Each data point represents the level of gene expression by individual mice in each group as determined by RT-PCR and normalized to the mean value of the PBS-vaccinated group. The bars represent median values for each group and Mann-Whitney P values for two-group comparisons are displayed in the figure. Statistical comparisons of the variances of the medians of the three groups were: IL-12b, P = 0.0006; IFN-γ, P = 0.0033; IL-4, P = 0.0702; IL-21, P = 0.0011; IL-6, P = 0.0033; and RANTES, P = 0.0112, Kruskal-Wallis test.

The cytokine milieu after vaccination influences the differentiation and polarization of naïve CD4+ T cells. IFN-γ, IL-12, and RANTES (CCL5) promote a Th1 response, IL-4 a Th2 response, IL-6 a Th17 response, and IL-21 a Tfh (T follicular helper) response [41]–[44]. To determine whether BCG and 3dBCG induce different patterns of expression of cytokines that may explain some of the differences in adaptive immunity reported above, we inoculated C57Bl/6 mice intravenously with 1.5×10^7^ CFU of BCG, 3dBCG, or PBS (control). Spleens were harvested 72 hours later and the mRNA from whole spleens was evaluated by RT-PCR for these Th-polarizing cytokines. BCG- and 3dBCG-vaccinated mice exhibited increased expression of IFN-γ, IL-12p40, IL-6, and IL-21 compared to control mice (**Fig. 6**) whereas the expression of IL-4 was not significantly different in either vaccinated group or controls. The expression of some genes was significantly greater in 3dBCG- than BCG-vaccinated mice including 33% more IL-12p40 (IL-12b), 34% more IL-21, and 30% more RANTES, which was suppressed in BCG-vaccinated mice compared to the control and 3dBCG groups. In summary, within three days of inoculation, BCG and 3dBCG induce different patterns of expression of immune response genes likely to influence subsequent T cell responses.

### 3dBCG grows slower in vitro and persists less well in vivo

**Figure 7:**
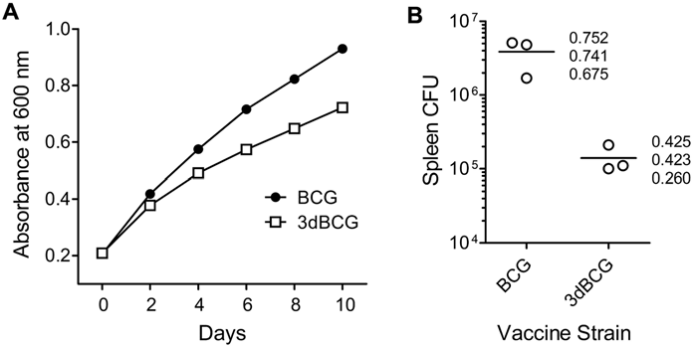
In vitro growth and in vivo persistance of BCG and 3dBCG. (A) Plot of the absorbance of cultures of BCG and 3dBCG during ten days of cultivation. A representative experiment shows A_600_ values every two days after Middlebrook 7H9 media containing Tween 80 in roller bottles was seeded with BCG or 3dBCG. The initial inoculum was adjusted to an A_600_ value of 0.2. (B) Spleen CFU at four weeks after intravenous inoculation of Balb/C mice with BCG or 3dBCG. Inocula were prepared based on absorbance values and then titered to determine the number of viable bacilli administered to each group of three mice – 6.6×10^6^ CFU for BCG; 7.7×10^6^ CFU for 3dBCG. The numbers represent the weights of the spleens in grams.

We have previously reported that under culture conditions wherein a detergent (*e.g*., Tween 80) is used to reduce the clumping of bacterial cells, antisense mutants of *M. tuberculosis* H37Rv with diminished production of SodA grow slower than the parent H37Rv strain [4]. Furthermore, these SodA mutants are attenuated in their ability to cause death and to persist in vivo [4]. Attenuation has also been reported for *secA2* deletion mutants of *M. tuberculosis* [5]. To determine whether the multiple mutations in antioxidant production introduced into BCG similarly affects in vitro growth and in vivo persistence, we monitored the absorbance of broth cultures of BCG and 3dBCG over time and compared the number of viable bacilli in the spleens of Balb/C at four weeks after intravenous inoculation. Relative to BCG, 3dBCG exhibited about 30% less of an increase in absorbance over ten days of cultivation **(** **Fig. 7A** **)**, indicating a modest reduction in its rate of growth. Furthermore, the number of 3dBCG recovered from the spleens of mice was reduced 32-fold compared to BCG **(** **Fig. 7B** **)**. The recovered bacilli represented 1.8 percent and 58.6 percent, respectively, of the number of bacilli inoculated (P = .027, two-sample T test). Thus, the stronger immune responses induced by 3dBCG compared to BCG as demonstrated above cannot be attributed to an effect of higher numbers of persisting vaccine bacilli. Instead the reduced persistence of 3dBCG in vivo is a consequence of the stronger immune responses induced by 3dBCG along with its slower rate of growth.

## Discussion

The capacity of *M. tuberculosis* and *M. bovis* to secrete multiple antioxidants may have evolved to enable growth under aerobic conditions by reducing oxidative damage to cell wall lipids and proteins, which comprise nearly 40 per cent of the dry weight of mycobacteria. However, beyond their role in microbial physiology, the production and secretion of large amounts of antioxidants gives the bacterium obvious advantages within mammalian hosts. Although best known for their microbicidal properties, oxidants produced by immune cells also help to coordinate the host response to infection. Oxidants have important signaling roles in the activation and apoptosis of macrophages [1], the maturation of dendritic cells [2], and the proliferation of T cells [3]. Chronic granulomatous disease (CGD), a genetic disease in which the phagocyte oxidase fails to assemble and produce superoxide, causes defects in signaling cascades that impair phagocyte activation [45], antigen presentation [46], and the development of memory lymphocytes [47]. By analogy, *M. tuberculosis* secretion of SodA, an enzyme that inactivates superoxide, may induce a similar albeit localized deficiency of superoxide that inhibits the development of adaptive immunity, resulting in chronic infection rather than eradication of the pathogen. Granulomatous pathology is a hallmark of both CGD and TB and the pathophysiology of inflammation in CGD involves impairment of a superoxide-dependent step in tryptophan metabolism [48]. In summary, the inactivation of host-generated oxidants by mycobacterial antioxidants may disrupt redox signaling during early infection and promote TB pathogenesis by weakening host immune responses and promoting tissue-damaging immunopathology.

By reducing antioxidant activity and secretion in BCG to yield 3dBCG, we unmasked immune responses during vaccination with 3dBCG that were suppressed by the parent BCG vaccine. Enhanced cytokine responses were detected within 3 days of vaccination. These differences may have clinical relevance as IL-12 and RANTES contribute to protection against TB in man [49], [50]. The early differences in cytokine responses were followed by differences in T cell responses. IL-12 and RANTES augment Th1 responses [41], [42] and IL-21 and IL-12 promote the expansion of CD8+ T cells [51], [52]. Thus the greater expression of IL-12p40, RANTES and IL-21 observed on day 3 post-vaccination in the 3dBCG group may have contributed to stronger antigen-specific IL-2+ production by CD4+ T cells as well as stronger CD8+ T cell responses. The enhanced CD8+ responses in 3dBCG-vaccinated mice may also have clinical relevance as CD8+ T cells contribute to protection against pulmonary TB in man [53], [54]. In effect, the modifications to BCG correct a deficiency in vaccine immunogenicity that may partly be responsible for the suboptimal efficacy of BCG against pulmonary TB.

Another deficiency of some current BCG vaccines is the poor induction of immune memory. The duration of protection conferred by vaccination with BCG in mice appears to be limited to the time that BCG persists in vivo [35]. However, persisting live vaccine bacilli represent a risk for disseminated BCG infection, especially in persons infected with HIV [31]. Thus, modifications to BCG that promote the development of immune memory and confer protection independent of persisting vaccine bacilli should improve the safety of BCG. In summary, the differences in the immune responses of 3dBCG- and BCG-vaccinated mice suggest that reducing the activity and secretion of microbial antioxidants in BCG leads to greater activation of innate immunity, stronger antigen-specific T cell responses, and better memory immunity. We suspect there are also other differences in response to these vaccines and this is a focus of ongoing investigation.

The enhanced immunogenicity of 3dBCG compared to BCG challenges the hypothesis that extensively cultivated BCG daughter strains are over-attenuated [32], [33]. To briefly review the background of the over-attenuation hypothesis and its influence on BCG vaccination practices, as early as the late 1940s concerns were raised about the possible over-attenuation of BCG [55]. The context was the observation of highly variable protection, from 0% to 80%, against pulmonary TB in human studies of BCG vaccination [13], [14]. Furthermore, investigations in mice indicated that BCG daughter strains differed in their ability to persist in vivo and to confer protection against challenge by virulent *M. tuberculosis* [27], [56], [57]. Indeed, protection in mice correlated directly with the invasiveness and persistence of the BCG daughter strain [57]. Such observations, along with the assumption that virulence would be lost rather than gained during cultivation in vitro, led to the conclusion that virulent BCG substrains should be preferred as they were the most like the original protective vaccine [30] that had exhibited 80% protective efficacy against pulmonary TB in uncontrolled studies of nursing and medical students in the 1920s [58], and also in an early controlled clinical trial in the 1930s [59].

The opinion that virulent BCG daughter strains were the most like the original vaccine influenced the selection of the relatively virulent BCG Danish 1331 and BCG Pasteur 1173P2 vaccines for the large controlled trial of vaccination with BCG in the Chingleput region of South India between 1968 and 1971 [13], [60], [61]. However neither vaccine was effective in persons over 14 years of age. Although this failure is often attributed to a confounding effect by environmental mycobacteria found in tropical climates [14], this explanation ignores the fact that in an earlier trial in the Madanapalle region of South India, only 185 kilometers from Chingleput, BCG exhibited 60% protective efficacy with an annual rate (per 100,000) of 198 and 79 bacillary cases, respectively, among controls and BCG vaccinees between 25 and 45 years of age [62]. The protective vaccine used in the Madanapalle study came from a 1948 subculture of BCG Danish before it was freeze-dried in 1960 as BCG Danish 1331 [63]. Another clinical trial in the United Kingdom also used BCG Danish vaccine prepared between 1950 and 1952 to immunize 14 to 15 year-olds, finding a 77% reduction in pulmonary TB [64]. Thus, the clinical data indicate that at one time some BCG vaccines conferred protection against pulmonary TB in adolescents and adults, including tropical countries with a high prevalence of TB and environmental mycobacteria. However, as evidenced by the experience with BCG Danish, something happened during years of in vitro cultivation to make BCG become less effective against pulmonary TB. Yet as BCG Pasteur 1173P2 and Danish 1331 are relatively virulent BCG substrains [27], [30], [56], [61], their failure to protect against pulmonary TB at Chingleput and in subsequent case-control trials where other less virulent BCG daughter strains exhibited about 55% protective efficacy against pulmonary TB [65]–[67] should not be attributed to over-attenuation.

Apart from its failure to explain BCG's unreliable protection against pulmonary TB, the over-attenuation hypothesis is inconsistent with the observation that the “late” BCG vaccines Pasteur 1173P2 and Danish 1331 cause more adverse reactions in man and exhibit greater virulence in animal models than the “early” BCG Tokyo 172 vaccine strain [14], [28], [61], [68]. Although the greater virulence of late BCG daughter strains that were passaged more than 1000 times compared to an early BCG vaccine passaged only 172 times after it was brought to Japan by Kiyoshi Shiga in 1924 could be a coincidence of divergent evolution, observations involving vaccines representing “linear” evolution in the BCG Danish family similarly suggests that over time BCG became more virulent instead of more attenuated. In 1954, a few years after BCG Danish was the source of vaccine for the trials in Madanapalle and the United Kingdom in which protection against pulmonary TB was demonstrated, a freeze-dried seed lot from the 1077^th^ transfer of BCG Danish was preserved as BCG London/Glaxo [63]. Six years later BCG Danish 1331 was preserved [63]. Yet despite its later derivation, BCG Danish 1331 is more virulent than BCG London/Glaxo in animal models. In one study the intraperitoneal injection of a 5 mg dose of Danish 1331 killed male golden hamsters within 100 days whereas more than 50% of hamsters infected with the same amount of BCG London/Glaxo survived for more than 400 days [30]. Experiments to determine the minimum “sensitizing” dose, *i.e*., the lowest number of bacilli capable of inducing delayed type hypersensitivity in guinea pigs or bank voles show that it takes only 10 cfu of Danish 1331 compared to about 200 cfu of BCG London/Glaxo [61], [69]. Thus between transfer 1077 and transfer 1331, the BCG Danish family of vaccines became more invasive and lethal.

When combined with recent reports of the duplication and increased expression of genes encoding antioxidants in BCG [17], [19], our results suggest a new model to explain how BCG became more virulent in small animals and less effective in controlling pulmonary TB in man. Whereas reference isolates of *M. bovis* and BCG Tokyo 172 have a single copy of *sigH*, BCG Pasteur 1173P2 and Danish 1331 contain bacilli with duplicated or triplicated *sigH* [17]. Indeed, BCG Danish appears to be continuing to evolve such that whereas cells with either duplication or triplication of the DU2-III region of the chromosome that contains *sigH* coexist in Danish 1331, only the triplicated form was found in a BCG Danish strain grown continuously for 1,513 passages [17]. BCG Pasteur 1173P2 also exhibits three-fold higher expression of *sodA* than BCG Tokyo 172 and reference *M. bovis* [17]. In the context of our findings that SigH and SodA suppress host immune responses, these mutations suggest a mechanism by which BCG Pasteur 1173P2 and BCG Danish 1331 evolved to become more immune suppressive and thus more virulent than their predecessors. In vitro, a mutation that increases antioxidant production may benefit BCG by reducing oxidative damage to cell wall lipids that otherwise would yield aldehydes and other metabolites that retard growth. Presumably such BCG mutants could grow faster, and this may explain the increase in oxygen consumption and growth rate observed over time in the BCG Danish family [30] and also with BCG Phipps [70]. Our speculation that these increases in the rate of growth of BCG during decades of in vitro cultivation partly reflect mutations resulting in increased antioxidant production is consistent with our prior report that diminishing the production of SodA reduces the growth rate of *M. tuberculosis* [4] as well as our finding in this study that targeting the production of antioxidants in BCG to yield 3dBCG reduces the rate of growth. The practice of growing BCG aerobically with detergents to prevent clumping may have increased oxidant stress to cell wall structures and selected for increased antioxidant production. Then with each transfer the bacilli making more antioxidants represented a slightly greater proportion of the culture until they became dominant. In vivo, these mutations caused the vaccine to become less potent in activating host immunity. In effect, we believe that as BCG evolved it yielded daughter strains with an increased capacity for suppressing host immune responses.

The model of BCG becoming more immune suppressive may also provide insight into why the extensively cultivated BCG daughter strains have remained effective against disseminated TB while losing their ability to protect against pulmonary TB. As noted above, 3dBCG was better than the parent BCG vaccine at inducing CD8+ T cell responses, which may be crucial for killing *M. tuberculosis* within macrophages in the lung and thereby limiting the extent of tissue-damaging granulomatous inflammation. The immunologic mechanisms for controlling the dissemination of TB may be different from those needed to prevent lung destruction. Instead of requiring cytotoxic T cells, it seems that the dissemination of infection is controlled primarily with IFN-γ-producing CD4+ T cells that activate macrophages [71]. Paradoxically, clinical data suggest that the relatively virulent BCG daughter strains may be slightly more protective against disseminated TB in early childhood than the relatively immunogenic BCG daughter strains. For example, fewer cases of disseminated TB occurred in the first two years of life in children vaccinated with intradermal BCG Danish 1331 than with percutaneous BCG Tokyo 172 [72], despite the greater immunogenicity of BCG Tokyo [73] and its greater effectiveness against pulmonary TB [65]–[67]. The extensively cultivated BCG substrains persist longer in small animal models than BCG Tokyo 172 [28], [68] and in both mice and man persisting vaccine bacilli may help to maintain a population of IFN-γ-producing CD4+ T cells that can respond immediately to infection with *M. tuberculosis* to form granulomas and reduce dissemination. In contrast, as it typically takes several days for memory CD4+ lymphocytes to begin proliferation upon re-exposure to antigen [74], if the vaccine strain has mediated its own eradication by inducing more potent immune responses, there may be a slight delay in the CD4+ response.

Both early and late BCG daughter strains remain highly efficacious against TB meningitis and miliary TB in young children, and it is pulmonary TB that causes the greatest burden of disease globally [16]. Our results suggest it might be possible to make improvements in BCG that enhance protection against pulmonary disease in man. Molecular genetic techniques make it possible to reduce the activity of antioxidants below the level found in any BCG vaccine, past or present, and this may enable the construction of highly immunogenic BCG-derived vaccines that match or surpass the high protective efficacy against pulmonary TB observed in early clinical trials. Our findings demonstrate the value of targeting SigH and SodA in BCG. Other antioxidants that are more highly expressed in BCG daughter strains than *M. bovis* include thiol peroxidase, Rv3303c, and members of the whiB family of protein disulfide reductases [17]. If these antioxidants similarly suppress host immune responses they may also be promising targets for enhancing BCG immunogenicity.

Beyond the implications of these findings for single dose vaccination with BCG to prevent pulmonary TB, a more immunogenic BCG should be a better priming vaccine for subsequent boosting with a heterologous vaccine in accordance with new TB vaccination strategies [75]. A more immunogenic BCG also has implications for cancer as increasing host production of multiple cytokines with anti-tumor activity (*i.e*., IL-12p40, RANTES, IL21, and IL-2) has the potential to overcome some of the limitations of current BCG vaccines as cancer immunotherapy [76], [77]. Finally, BCG occupies a unique place in the vaccination schedule of infants throughout much of the world. Thus, a more immunogenic BCG may become a useful vector for expressing antigens of other pathogens including HIV and *Plasmodium* species to produce strong cell-mediated responses, including CD4+ helper responses, as part of vaccination regimens against AIDS, malaria, and other infectious diseases.

## Materials and Methods

### Bacterial isolates, plasmids, media, and growth conditions

Genetic tools and bacterial isolates are listed in **Table S1**. Genetic modifications were made to BCG Tice (Organon Teknika Corp., Durham, NC). Of note, although historically BCG Tice was considered to be a weak BCG vaccine that exhibited poor ability to persist in mice or confer protection against challenge with *M. tuberculosis* in mice [27], [57], it was reformulated with a subculture of BCG obtained from the Institut Pasteur in 1951 and subsequently BCG Tice has exhibited in vivo characteristics similar to BCG Pasteur 1173P2 [68], [78], [79].

*E. coli* strains were grown in LB media. To prepare vaccine inocula and to compare rates of growth, BCG Tice and modified strains were grown in roller bottles containing Middlebrook 7H9 media with 10% oleic acid-dextrose-catalase (OADC) enrichment supplemented with 0.2% glycerol, and 0.05% Tween80. Kanamycin (50 µg/ml or 25 µg/ml), apramycin (50 µg/ml), and hygromycin B (100 µg/ml or 50 µg/ml) were used to select colonies after genetic manipulations in *E. coli* or BCG, with higher concentrations for *E. coli* and lower concentrations for BCG.

### Molecular genetic manipulations

Plasmid and chromosomal integration vectors for allelic inactivation or expression of dominant-negative monomers of SodA were electroporated into BCG using standard methods [80]. After electroporation, the strains were incubated in Middlebrook 7H9 media with 5% CO_2_ at 37°C for 24 hrs before the suspension was selected on Middlebrook 7H11 agar containing antibiotics as needed. Successful transformation was confirmed by PCR of DNA unique to the vector.

The inactivation of *secA2* was performed using a two-step allelic exchange strategy with plasmid pMB179, as previously described in *M. tuberculosis* [5]. The inactivation of *sigH* was performed by using the cosmid-phasmid pYUB854-phAE87 system, containing the *hygR* cassette, to construct a specialized transducing phage [80]. Homologous recombination in BCG was predicted to eliminate the terminal 316 nucleotides of *sigH* including the region encoding a putative DNA binding motif. Southern hybridization of *DraIII*-digested DNA was used to verify allelic inactivation.

To construct dominant-negative (dn) enzyme monomers, *sodA* was PCR-amplified from DNA of *M. tuberculosis* strain H37Rv, ligated into pCR2.1-TOPO, and propagated in *E. coli* TOP 10. Site-directed mutagenesis was performed using primer overlap extension methods [81] and codon deletion was verified by DNA sequencing. The ΔH28ΔH76 mutant was verified to lack superoxide dismutase (SOD) activity by its inability to complement the growth of the SOD double-negative (*sodA-, sodB-*) *E. coli* strain CK9C1891 [82]. The dn-*sodA* open reading frame was then ligated behind DNA from a 341-bp region upstream of isocitrate lyase (*icl*) from H37Rv, a macrophage-inducible promoter [83], in an effort to not severely impair the growth of SodA-diminished strains in vitro while reducing enzyme activity in vivo. Finally, alleles encoding dominant-negative enzyme monomers were ligated into shuttle plasmid or chromosomal integration vectors.

### Assays of SodA quantity and activity

Immunoblotting was used to compare SodA enzyme quantity. Bacterial lysates were adjusted to a standard A280 value, applied to a SDS-12% PAGE gel for separation of proteins by electrophoresis and transferred to nitrocellulose membranes and hybridized with a 1∶1000 dilution of rabbit polyclonal antisera against recombinant SodA [84]. To measure superoxide dismutase activity in lysates and supernatants of vaccine cultures, we monitored the % inhibition of a superoxide anion (O_2_^−^) - induced reduction of a water-soluble tetrazolium (WST-1) salt to a formazan dye by colorimetric methods (SOD Assay Kit-WST, Dojindo Molecular Technologies, Inc., Gaithersburg, MD). One unit was defined as the amount of SodA that inhibited color development at 440 nm by 50%.

### Mice and Infections

Experiments in mice were approved by the Vanderbilt Institutional Animal Care and Use Committee and conform to regulatory standards for research involving vertebrate animals. Female C57Bl/6 mice and Balb/c mice aged 5–6 weeks were purchased from Jackson Laboratories (Bar Harbor, ME). To assess persistence of the vaccine strain in vivo, mice were inoculated retro-orbitally with approximately 5×10^6^ CFU. To assess immune responses, mice were vaccinated subcutaneously or retro-orbitally with sterile broth media or with preparations of BCG or 3dBCG (BCGΔ*secA2*Δ*sigH*dnSodA) in broth media at 5×10^5^ CFU. In order to eliminate the vaccine strain before a mycobacterial challenge, mice were treated with isoniazid and rifampin by adding 100 mg/liter of each antibiotic to drinking water [85]. After 30 days on this regimen, mice were then rested for at least two weeks before intravenous challenge (retro-orbital or tail-vein) with 2×10^7^ CFU of BCG Tice.

### Gene expression analysis

C57Bl/6 mice, six per group, were injected retro-orbitally with 1.5×10^7^ CFU of BCG, 3dBCG, or phosphate-buffered saline (control). Mice were euthanized 72 hours later and the spleens were harvested and immersed in liquid nitrogen. To recover total cellular RNA, each spleen was homogenized with a mortar and pestle in the presence of liquid nitrogen. The homogenized tissue was extracted with Trizol (Gibco/Invitrogen, Carlsbad, CA) followed by a chloroform extraction and isopropanol precipitation. The pellet was resuspended in DNase/RNase free water (Gibco/Invitrogen, Carlsbad, CA) and the sample was DNase-treated (Promega, Madison, WI) for 30 minutes at 37°C. Total cellular RNA was further purified with a Qiagen RNeasy column purification system (Qiagen, Valencia, CA), quantitated with a spectrophotometer, and normalized. cDNA was prepared from each RNA sample with the High Capacity cDNA Reverse Transcription Kit (Applied Biosystems, Foster City, CA) and analyzed with Taqman primer-probe pairs (Applied Biosystems) on an Applied Biosystems 7900HT Real-Time PCR system.

### Measurement of Immune Reponses

To obtain lymphocytes for measurement of immune responses after vaccination or challenge, mice were euthanized and splenocytes were isolated and suspended in RPMI, enumerated, and three million cells were used for each stimulation condition. Synthetic peptides TB10.4_74-88_ - ST15, a CD4 epitope^29^ and TB10.3/10.4_20-28_ - GL9, a CD8 epitope^30^ (Genemed Synthesis Inc, San Antonio, TX) and BCG-infected bone-marrow derived macrophages (BMDMs) were used as stimulants. BMDMs were derived from femur bone marrow stem cells which were cultured for 5 days in petri-dishes in DMEM medium containing 10% fetal calf serum and 20% L-929 conditioned medium. Three days before harvesting splenocytes, the BMDMs were transferred to 24-well plates at 1 million cells per well, and infected the next day with 5∶1 MOI BCG Tice in the presence of 20 ng/ml IFN-γ for 4 hours. Paired wells of BMDMs were similarly treated with IFN-γ but left uninfected. Forty-eight hours after infection of BMDMs, 3 million harvested splenocytes were re-stimulated on uninfected and BCG-infected BMDMs for 20 hours, the last 18 hours in the presence of brefeldin A. For measurement of cytokine responses to peptide, harvested splenocytes were stimulated with or without peptides for 6 hours, the last 4 hours in the presence of brefeldin A. Cytokine production was assessed by intracellular cytokine staining and flow cytometry.

### Intracellular cytokine staining and flow cytometry

Stimulated splenocytes were stained sequentially with live/dead fixable dead cell stain kit - violet fluorescent reactive dye (Invitrogen Molecular Probes, Carlsbad, CA) and a cocktail of surface antibodies - anti -CD3 APC-Cy7, -CD4 PE-Texas Red, and -CD8 Pacific Orange (Invitrogen), fixed and permeabilized with cytofix/cytoperm solution and stained with anti -IFN-γ PE-Cy7, -TNF-α FITC, and –IL-2 PE (all from BD biosciences, San Jose, CA unless otherwise indicated) and approximately 1,000,000 events within the lymphocyte gate were acquired using a FACSAria and analyzed with FacsDIVA software (BDbiosciences, San Jose, CA). The total number of cytokine+ CD4+ and CD8+ T cells per whole spleen was calculated from the products of the total number of splenocytes multiplied by the proportion of cytokine+ CD4+ and CD8+ T cells under each stimulation condition. Antigen-specific cytokine+ T cells were calculated as the difference between cytokine+ cells from the unstimulated and stimulated conditions.

### Statistics

The planned comparison in each experiment was between the immune response to BCG and the immune response to 3dBCG. Unless otherwise indicated, these comparisons were performed using the Mann-Whitney determination for a non-parametric distribution, two-tailed, and a P value of less than 0.05 was considered significant. Secondary comparisons involved the responses of each vaccinated group of mice to the responses of unvaccinated mice. To compare the medians of all three groups we used the Kruskal-Wallis non-parametric test and the P values for the significance of the variance of the three medians are noted in the Figure legends. Comparisons between the immune responses in a vaccinated group and the unvaccinated group were further analyzed using the Mann-Whitney test, two-tailed, and a P value of less than 0.05 was considered significant. Correlation coefficients were calculated using non-parametric Spearman calculations. Statistical analyses were performed using Prism 5.0 software (GraphPad) and only significant (P<0.05) or close to significant (P<0.1) values are indicated.

## Supporting Information

Table S1 Tools for genetic manipulations and bacterial strains
(0.05 MB DOC)
