# Supplementary material for: Reducing the Activity and Secretion of Microbial Antioxidants Enhances the Immunogenicity of BCG
Source: PLoS One. 2009 May 13;4(5):e5531. doi: 10.1371/journal.pone.0005531 (PMC2677452; doi:10.1371/journal.pone.0005531)
Supplement: Table S1 — Tools for genetic manipulations and bacterial strains (0.05 MB DOC) [file pone.0005531.s001.doc]

**Table S1. Tools for genetic manipulations and bacterial strains**

| **Name** | **Description** | **Reference or source** |
| --- | --- | --- |
| **Plasmids** | | |
| pCR2.1-TOPO | Plasmid for cloning PCR products | Invitrogen Corp., Carlsbad, California |
| pBC SK+ | *E. coli* phagemid vector | Stratagene, La Jolla, CA |
| pLUC10 | *E. coli*-mycobacterial shuttle plasmid containing firefly luciferase gene | Robert Cooksey, CDC, Atlanta, Georgia [1] |
| **Chromosomal integration vectors** | | |
| pMP399 | *E. coli*-mycobacterial *attB* integration vector containing aacC41 gene encoding apramycin resistance | Martin Pavelka, University of Rochester, NY [2] |
| pMP399-dn*sodA*-ΔH28ΔH76 | pMP399 with ΔH28ΔH76 dn mutant *sodA* allele behind *aceA* (*icl*) promoter – the mutant *sodA* also contains G🡪S substitution at C-terminus | This study |
| **Allelic inactivation tools for chromosomal genes** | | |
| pMB179 | Suicide vector carrying an in-frame unmarked *secA2* deletion allele, counterselectable *sacB*, and selectable hygromycin resistance (hygR) marker | [3] |
| pYUB854 | Cosmid containing hygR cassette and multiple cloning sites flanking the cassette to allow directional cloning | William Jacobs, Jr., Albert Einstein College of Medicine, NY [4] |
| phAE87 | Conditionally replicating shuttle phasmid for chromosomal gene inactivation in mycobacteria | William Jacobs, Jr. [4] |
| pYUB854-*sigH* | Cosmid containing *sigH* flanking DNA for allelic inactivation | This study |
| **Strains** | | |
| TOP 10 | *E. coli* host strain for cloning PCR products, used in combination with pCR2.1-TOPO | Invitrogen Corp., Carlsbad, California |
| DH5α | *E. coli* host strain for genetic manipulation, construction of mutant enzyme expression vectors | Life Technologies, Gaithersburg, MD [5] |
| CK9C1891 | *E. coli* (*sodA-, sodB-*) used to assess activity of SodA mutants | Danièle Touati, Institut Jacques Monod, Paris [6] |
| HB101 | *E. coli* host strain for genetic manipulation, used as host for pYUB854 cosmid | Promega Corp., Madison, Wisconsin |
| mc^2^155 | Transformation-efficient strain of *Mycobacterium smegmatis* | William Jacobs, Jr., Albert Einstein College of Medicine [4,7] |
| BCG Tice | Bacillus Calmette-Guérin, substrain Tice | Organon Teknika Corp., Durham, NC [8–10] |
| **1^st^ generation mBCG vaccines (one modification)** | | |
| BCGdnSodA | BCG containing pMP399-dn*sodA*-ΔH28ΔH76 | This study |
| BCGΔ*secA2* (MB546) | BCG with allelic inactivation of *secA2* | This study |
| BCGΔ*sigH* | BCG with allelic inactivation of *sigH* | This study |
| **2^nd^ generation mBCG vaccines (two modifications)** | | |
| DDBCG | “double-deletion BCG”, with allelic inactivation of *secA2* and *sigh* | This study |
| BCGΔ*sigH*dnSodA | BCGΔ*sigH* containing pMP399-dn*sodA*-ΔH28ΔH76 | This study |
| BCGΔ*secA2*dnSodA | BCGΔsecA2 containing pMP399-dn*sodA*-ΔH28ΔH76 | This study |
| **3^rd^ generation mBCG vaccine (three modifications)** | | |
| 3dBCG | DDBCG containing pMP399-dn*sodA*-ΔH28ΔH76 | This study |

1. Cooksey RC, Crawford JT, Jacobs WR, Jr., Shinnick TM (1993) A rapid method for screening antimicrobial agents for activities against a strain of *Mycobacterium tuberculosis* expressing firefly luciferase. Antimicrob Agents Chemother 37: 1348-1352.

2. Consaul SA, Pavelka MS, Jr. (2004) Use of a novel allele of the Escherichia coli aacC4 aminoglycoside resistance gene as a genetic marker in mycobacteria. FEMS Microbiol Lett 234: 297-301.

3. Braunstein M, Espinosa BJ, Chan J, Belisle JT, Jacobs WR, Jr. (2003) SecA2 functions in the secretion of superoxide dismutase A and in the virulence of *Mycobacterium tuberculosis*. Mol Microbiol 48: 453-464.

4. Braunstein M, Bardarov SS, Jacobs WR, Jr. (2002) Genetic methods for deciphering virulence determinants of *Mycobacterium tuberculosis*. Methods Enzymol 358: 67-99.

5. Hanahan D (1983) Studies on transformation of *Escherichia coli* with plasmids. J Mol Biol 166: 557-580.

6. Carlioz A, Touati D (1986) Isolation of superoxide dismutase mutants in *Escherichia coli*: is superoxide dismutase necessary for aerobic life? EMBO J 5: 623-630.

7. Snapper SB, Melton RE, Mustafa S, Kieser T, Jacobs WR, Jr. (1990) Isolation and characterization of efficient plasmid transformation mutants of Mycobacterium smegmatis. Mol Microbiol 4: 1911-1919.

8. Dubos RJ, Pierce CH (1957) Tice strain of BCG. Am Rev Tuberc 75: 692-693.

9. Dubos RJ, Pierce CH (1956) Differential characteristics in vitro and in vivo of several substrains of BCG. I. Multiplication and survival in vitro. Am Rev Tuberc 74: 655-666.

10. Oettinger T, Jorgensen M, Ladefoged A, Haslov K, Andersen P (1999) Development of the *Mycobacterium bovis* BCG vaccine: review of the historical and biochemical evidence for a genealogical tree. Tuber Lung Dis 79: 243-250.
